# The Effect of Low CD4+ Lymphocyte Count on the Radiographic Patterns of HIV Patients with Pulmonary Tuberculosis among Nigerians

**DOI:** 10.1155/2013/535769

**Published:** 2013-01-30

**Authors:** Christopher Affusim, Vivien Abah, Emeka B. Kesieme, Kester Anyanwu, Taofik A. T. Salami, Reuben Eifediyi

**Affiliations:** ^1^Department of Family Medicine, Ambrose Alli University, PMB 8, Ekpoma, Edo state, Nigeria; ^2^Department of Family Medicine, University of Benin Teaching Hospital, Benin, Edo State, Nigeria; ^3^Department of Surgery, Ambrose Alli University, PMB 8, Ekpoma, Edo state, Nigeria; ^4^Department of Internal Medicine, Ambrose Alli University, PMB 8, Ekpoma, Edo state, Nigeria; ^5^Department of Obstetrics and Gynaecology, Ambrose Alli University, PMB 8, Ekpoma, Edo state, Nigeria

## Abstract

*Objective*. To assess the radiographic features in patients with Human Immunodeficiency Virus (HIV) complicated by pulmonary tuberculosis (PTB), and the association with CD4 lymphocyte count and sputum smear. *Method*. A prospective study was carried out on 89 HIV positive patients with PTB. The demographics, smoking history, sputum smear result, chest radiographic findings and CD4 lymphocyte count were documented. *Results*. Out of the 89 patients recruited in the study, 41 were males and 48 were females. Eighteen (18) patients had typical radiographic features, 60 patients had atypical radiographic features while only 11 of them had normal radiographic films. Sixty eight (68) patients had CD4 count <200 cells/mm^3^, 19 patients had CD4 count between 200–499 cells/mm^3^, while only 2 patients had CD4 count from 500 cells/mm^3^ upwards. The association between low CD4 count and radiographic finding was statistically significant, (*P* value <0.05). Sixty (60) patients had negative sputum smear for Acid and Alcohol Fast Bacilli (AAFB), while the remaining 29 patients had positive smear. The association between low CD4 count and negative smear was statistically significant (*P* value <0.05). *Conclusion*. The radiographic pattern and the result of the sputum smear for AAFB has a significant relationship and association with the immune status of patients with Human Immunodeficiency Virus (HIV) complicated by pulmonary tuberculosis.

## 1. Introduction

Human immunodeficiency virus (HIV) is a potent risk factor for tuberculosis (TB), both through an increase in the reactivation of the latent *Mycobacterium tuberculosis* infection and through an accelerated progression from infection to active disease, by undermining the cell-mediated immunity through depletion of CD4 lymphocytes [1–4]. 

TB has a great impact on morbidity and mortality in HIV-1 infected individuals than all other opportunistic infections [3]. TB and HIV infections have a synergistic influence on the host immunoregulation. TB can develop at any stage of immunosuppression regardless of the level of the circulating CD4+ T-lymphocytes [4]. CD4+ lymphocytes count is one of the surrogate markers for evaluating the degree of immunosuppression and HIV disease progression [4].

The levels of circulating CD4+ lymphocytes has a great impact on the radiographic pattern of TB. In HIV infections, TB can produce both typical and atypical radiographic patterns depending on the degree of immunosuppression [5–8]. Atypical radiographic presentations are lower frequency of cavitations, higher frequency of mediastinal lymphadenopathy, lower lung zone infiltrates, and even a normal chest radiograph. Typical presentations include upper lobe fibrosis, bilateral infiltrates, consolidation, and cavitations [6–8]. Thus patients with low CD4 lymphocyte count have more of features of primary TB, while those with a high CD4 count will have features of postprimary TB.

This study was undertaken to determine the effect of low CD4+ lymphocyte count on the radiographic patterns of HIV patients with pulmonary TB among Nigerians. There is dearth of studies on this subject in Nigeria.

## 2. Materials 

### 2.1. Study Setting

 The study was done at University of Benin Teaching Hospital from April to May 2007. This hospital is a government designated centre of excellence for the management of HIV/AIDS. The patients for the study were recruited from the HIV clinic of this hospital. They were confirmed HIV positive patients with pulmonary TB.

### 2.2. Inclusion Criteria

1. HIV positive patients with pulmonary TB aged 18 years and above who presented at the clinic within the study period and who had not commenced drug treatment.
2. All HIV positive patients with pulmonary TB who consented to join in the study.

### 2.3. Exclusion Criteria

1. Patients with indeterminate PTB.
2. Patients with other immunosuppressive diseases like diabetes mellitus, malignancies, and so forth.

## 3. Method of Data Collection 

This was a prospective study done from April to May 2007. Informed consent was obtained from the patients. Information was gathered from the patient's case note. The subject's biodata and the results of the following investigations were collected.

- Chest radiograph.
- Sputum for acid and alcohol fast bacilli (AAFB).
- CD4 cell count.

The chest radiographs were blindly reported by two radiologists who were not aware of the enrolment into the study. The two reached a consensus on radiological findings of a radiograph when there is a disagreement in their evaluation. They evaluated the radiographs for mediastinal lymphadenopathy, infiltrates, cavitations, pleural effusions, and localized or military shadows and also determined the predominantly affected lung zones.

The chest radiograph results were grouped into three namely, those with typical features, those with atypical features, and those with normal films. The sputum smear result was grouped into two namely, those positive for AAFB and those negative for AAFB. The CD4 lymphocyte cell count results was also grouped into three namely, CD4 count <200 cells/mm^3^, CD4 count 200–499 cells/mm^3^, and CD4 count from 500 cells/mm^3^ upwards. 

## 4. Method of Data Analysis

 The statistical package for social sciences (SPSS) version 16.0 was used to record and analyse the data. Frequency tables were drawn to show the distribution of data within variables. Contingency tables were drawn to compare two discrete variables. Pearson Chi-square was used to test significance. A *P*  value < 0.05 was considered significant.

## 5. Case Definition

 The diagnosis of PTB/HIV coinfection was based on criteria for diagnosing TB in poor resource settings where there are no facilities and manpower for *mycobacterium tuberculosis* culture: (a) the diagnostic criteria of TB given in the World Health Organisation (WHO) treatment of tuberculosis guideline for national programmes [5]; (b) specificity of clinical criteria in diagnosing TB patient [6, 9].

## 6. Results

A total of 89 HIV positive patients with pulmonary TB were recruited into the study. Their ages range from 19 years to 65 years. The mean age was 37.73 years. There were 41 males (46.07%) and 48 females (53.93%). Forty-six (46) patients (51.68%) were married, 31 patients (34.83%) were single, and 2 patients (2.25%) were separated from their spouses, while 10 patients (11.23%) had lost their spouses. Five (5) patients (5.62%) had no formal education, 17 patients (19.10%) had tertiary education, and 40 patients (44.94%) had secondary education, while 27 patients (30.34%) had primary education.

More of the patients (74 patients (83.15%)) had never smoked, while a small number (15 patients (16.85%)) had smoked at one time or the other in their lives. Sixty (60) patients (67.42%) had negative sputum smear for acid and alcohol fast bacilli, while 29 patients (32.58%) had positive smear.

The chest X-ray results were as follows: 18 patients (20.22%) had typical chest X-ray features, and 60 patients (67.42%) had atypical features, while the least number 11 patients (12.36%) had normal features. The mean CD4 lymphocyte cell count was 125 cells/mm^3^, 68 patients (76.40%) had CD4 count less than 200 cells/mm^3^, and 19 patients (21.35%) had CD4 count levels between 200 and 499 cells/mm^3^, while 2 patients (2.25%) had CD4 cell count from 500 cells/mm^3^ upwards.  Table 1 shows the characteristics of the patients studied.

All the 11 patients who had normal chest X-ray findings had CD4 count less than 200 cells/mm^3^, while the remaining with CD4 count more than 200 cells/mm^3^ had either atypical or typical findings. The association between severe immunosuppression and normal radiograph was statistically significant (*P*  value < 0.05) (Table 2). 

Out of all the patients with CD4 less than 100 cells/mm^3^, a total of twenty-two (22) patients (24.7%) had CD4 count below 50 cells/mm^3^, while sixteen (16) patients (17.98%) had CD4 count between 50 and 100 cells/mm^3^. Eighteen (18) of those with CD4 count <50 cells (81.81%) had atypical X-ray features, while fourteen (14) of those with CD4 count 50–100 cells (87.5%) also had atypical X-ray features.

Out of the 60 patients that had negative sputum smears, only 2 of them had CD4 count ≥200 cells/mm^3^. The other 58 patients with negative smears had CD4 count <200 cells/mm^3^, and the association between severe immunosuppression (CD4 count <200) and negative sputum smear was significant (*P*  value < 0.05) (Table 3).

## 7. Discussion

Results from the study showed a significant relationship between the CD4 lymphocyte cell count and the radiographic features of HIV positive patients with pulmonary tuberculosis (Table 2). The CD4 count is an indicator of immune status and stage of HIV infection. Severe immunosuppression and CD4 count <200 cells/mm^3^ were significantly associated with the presence of mediastinal lymphadenopathy. This is in keeping with other studies worldwide [9–11]. Other features of primary TB (atypical features) like middle and lower lung zone involvement, military pattern, and normal films were also more common in patients with CD4 count <200 cells/mm^3^, (Table 2). 

Cavity formation and other features of postprimary TB on chest radiograph were found to be common and significantly associated with more immunocompetency (CD4 count ≥200 cells/mm^3^). Several other studies on the association between cavity formation and high CD4 count confirm this [11, 12]. Formation of cavities in TB infection requires an adequate delayed type of hypersensitivity reaction and an intact cell-mediated immunity in the host. Typical features (upper lung zone involvement) are seen more often in HIV patients with less immunosuppression than in those with severe immunosuppression. It is not surprising that HIV patients with high CD4 counts will have upper lung zone involvement because TB in the upper lung zone is usually common in HIV negative patients with high CD4 count and thus immunocompetent. 

Only 11 out of the 89 patients in the study had normal chest radiographs, and all the 11 patients had CD4 count <200 cells/mm^3^. The association between severe immunosuppression and normal radiograph was significant (Table 2). This was supported by another study [13] but was not supported by a study done in USA which found no significant association between CD4 count and normal chest radiograph [11]. 

The finding of a normal chest radiograph and negative sputum smear microscopy, in HIV patients coinfected with pulmonary TB, poses a great challenge for the diagnosis of pulmonary TB in poor resource countries, where facilities for culture of *mycobacterium tuberculosis* are scanty or nonexistent, and thus diagnosis requires high index of suspicion. More studies are required to confirm if the absence of radiographic findings represents early stages of either primary TB or a reactivation, or one caused by intrathoracic lymphadenopathy unable to be detected by simple radiographic examination [11, 12]. In a cross tabulation between sputum smear and chest X-ray findings, it was found that most of the patients that had atypical chest X-ray features also had negative sputum smears (Table 4). The relationship between these two was significant. This finding confirms that in severe immunosuppression, the usual immune response to TB infection is no longer maintained.

In this study, atypical or primary pattern was common in patients with CD4 count <200 cells/mm^3^, while typical or postprimary pattern was commoner in those with CD4 count >200 cells/mm^3^ and commonest in those with CD4 count ≥500 cells/mm^3^. This was similar to what was found in some other studies [10, 11]. The different radiographic appearances have different pathogenesis, and these were greatly modified by the level of CD4 count (degree of immunosuppression) and cell-mediated immunity. 

This study has revealed that various radiographic manifestations of HIV coinfected with pulmonary TB are related to the level of immunosuppression (CD4 count). Physicians need to be aware of this finding, and those in countries with poor resources should have high index of suspicion to be able to make proper diagnosis.

## Figures and Tables

**Table 1:** Characteristics of the patients in the study.

Characteristics of the patients	Frequency	Percentage
Age	Age range: 19–65 years	
	Mean age: 37.73 years	

Sex	Males: 41	46.07%
	Females: 48	53.93%

Marital status	Married: 46	51.68%
	Single: 31	34.83%
	Separated: 2	2.25%
	Widowed: 10	11.23%

Smoking history	Have smoked: 15	16.85%
	Never smoked: 74	83.15%

Sputum smear	Positive: 29	32.58%
	Negative: 60	67.42%

	Typical features: 18	20.22%
Chest X-ray	Atypical features: 60	67.42%
	Normal features: 11	12.36%

	<200 cells/mm^3^ − 68	76.40%
CD4 cell count	200–499 cells/mm^3^ − 19	21.35%
	500 cells/mm^3^ and above− 2	2.25%

**Table 2:** Relationship between levels of CD4 count and chest X-ray features.

CD4 count	Chest X-ray features		
	Typical	Atypical	Normal
<200	2	55	11
200–499	14	5	0
≥500	2	0	0

Total	18	60	11

*P* value < 0.05. Relationship is significant.

**Table 3:** Relationship between levels of CD4 count and sputum smear.

CD4 count	Sputum smear	
	Positive	Negative
<200	10	58
200–499	17	2
≥500	2	0

Total	29	60

*P* value < 0.05. Relationship is significant.

**Table 4:** Relationship between sputum smear and chest X-ray features.

Sputum smear	Chest X-ray features		
	Typical	Atypical	Normal
Positive	16	12	1
Negative	2	48	10

Total	18	60	11

*P* value < 0.05. Relationship is significant.
